# Changing the Surgical Approach to Breast Diseases During the COVID-19 Pandemic Period

**DOI:** 10.7759/cureus.45653

**Published:** 2023-09-20

**Authors:** Huda Umit Gur, Mahmut Said Degerli

**Affiliations:** 1 General Surgery, Haseki Training and Research Hospital, University of Health Sciences, Istanbul, TUR; 2 General Surgery, Bakirkoy Dr. Sadi Konuk Training and Research Hospital, University of Health Sciences, Istanbul, TUR

**Keywords:** surgical approach, pandemic hospital, breast diseases, breast cancer, covid-19

## Abstract

Background

Postponing elective surgeries during the COVID-19 pandemic has also affected the approach to both malignant and benign breast diseases. This paper aims to share how the COVID-19 pandemic affects our approach to breast cancer, benign breast cases, and the procedures' results.

Methodology

A cross-sectional study was conducted in a tertiary-level public hospital in Istanbul, Turkey. We retrospectively analyzed our treatment options for patients diagnosed with breast cancer and benign breast disease in the general surgery clinic of a tertiary hospital that declared a pandemic status between March 11, 2020, and June 1, 2020.

Results

The number of patients who visited the breast outpatient clinic and received a diagnosis of breast cancer was 23. Among the benign diseases, no intervention was made except for abscess (eight patients, 40%) and mastitis (12 patients, 60%).

Conclusions

Patients with acute abscesses and mastitis were treated for both diagnostic and therapeutic purposes. Chemotherapy and hormone therapy were chosen for those diagnosed with cancer. Priority was given to oncology protocols rather than surgical approaches during the pandemic. We think that different approaches will be defined as the pandemic continues.

## Introduction

In December 2019, the first COVID-19 outbreak in Wuhan, province of China, caused devastating global impacts in a short time. The first case was reported in Turkey on March 11, 2020 [1]. In the three months in between, several publications have emerged around the world about the measures to be taken during the pandemic, the operations to be done or postponed, and alternative treatments [2-4]. Most of them suggested postponing operations performed under elective conditions. Recommendations about emergency operations and approaches were made in these publications [5,6].

The COVID-19 pandemic became one of the biggest threats to all humanity. Hospital beds and protective equipment were needed against thousands of deaths. Surgical algorithms were crucial to protect uninfected patients and staff from extra viral exposure and in-hospital transmission [7].

The American College of Surgeons (ACS) promotes breast-conserving surgery as much as possible in breast cancer cases. They also recommend reconstruction (autologous and implant) after mastectomy. However, with the COVID-19 pandemic, reconstruction procedures were postponed, and operations were limited to patients at risk of death if not operated on urgently [8].

The COVID-19 pandemic is still ongoing, and many publications and reviews are emerging regarding breast cancer [8]. There are also publications about breast cancer on the normalization process [9-10]. However, there have been no suggestions about follow-up and treatment in English literature related to benign breast diseases such as fibroadenomas, papillomas, lesions diagnosed with adenosis, and granulomatous mastitis during the pandemic. This study aims to share our changing surgical approaches during the COVID-19 pandemic.

## Materials and methods

Our hospital became a pandemic-designated facility on March 11, 2020. The first case of COVID-19 in Turkey was admitted to our hospital. All elective cases were postponed, and only urgent and life-threatening conditions were treated with surgery. Until June 1, 2020 (the start of the normalization process in Turkey), treatment was planned for 23 patients with newly diagnosed breast cancer, and eight (34.7%) received neoadjuvant treatment. Surgery was performed on 20 patients with benign breast diseases. The evaluated parameters included demographic characteristics, comorbidities, indications for surgery, preoperative patient assessment environment, preoperative imaging methods, indications for surgery, surgical procedures, anesthetic procedures, the status of postoperative suspicion of COVID-19, and postoperative morbidity and mortality rates.

Approval 

Board of Ethics approval for this study was obtained from the Ethics Commission of Haseki Training and Research Hospital, Health Sciences University (approval no. 156-2020; approval date July 08, 2020). 

Statistical methods

Descriptive statistics included mean, standard deviation, median, minimum, maximum, frequency, and ratio. IBM SPSS Statistics for Windows, Version 20.0. (IBM Corp., Armonk, NY, USA) was used for analyses.

## Results

**Figure 1:**
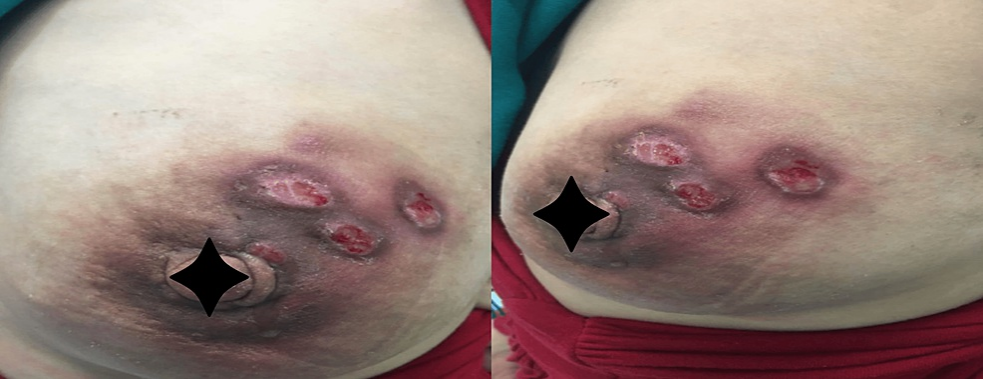
Patient with granulomatous mastitis.

**Figure 2:**
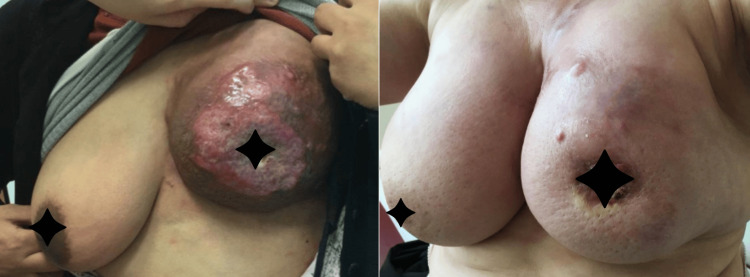
Patients with locally advanced breast cancer.

Hormonotherapy protocol was preferred for hormone receptor-positive breast cancer patients. The mean age of this group of patients was 69 years (ranging between 61 and 79 years). Aromatase inhibitors were preferred for all postmenopausal patients. Tumor size was at most 4 cm (1.8-4 cm), and axilla biopsy was positive in three patients, negative in three patients, and suspicious in one patient. The Ki67 index ranged from 10% to 35%. Metastasis screening, conducted with positron emission tomography-computed tomography (PET-CT) for all patients, yielded negative results for the entire body. The follow-up of tumor size in patients was conducted monthly through physical examination and ultrasound (Table 1). Of the eight patients in whom the neoadjuvant chemotherapy protocol was initiated, three had more than one malignant mass (multi-focal), and seven patients had biopsy-verified axillary positivity. Tumor size ranged from 1.7 to 5 cm. Metastasis screening was negative for the whole body with PET-CT for each patient. Standard neoadjuvant chemotherapy was started for our patients. During this, none of the patients completed the chemotherapy protocol (Table 2). Four patients, consisting of two with stage 1 tumors and two with stage 2 tumors, all of whom were axilla negative, underwent breast conservation surgery and sentinel lymph node biopsy. One patient had a mastectomy and axillary lymph node dissection. The patients whose neoadjuvant therapy was completed (treatment began before the pandemic) were operated on within six to eight weeks. Modified radical mastectomy procedures were performed in two patients. Segmental mastectomy and sentinel lymph node biopsy were done on two patients, and axillary cure was performed in one patient with segmental mastectomy. The oncoplastic surgical approach was not preferred in any patient (Table 3). Interventional radiological procedures were performed on five (25%) patients with puerperal mastitis, eight (40%) patients with puerperal abscess, and seven (35%) patients with undiagnosed granulomatous mastitis. However, surgical abscess drainage was applied to three (15%) patients with granulomatous mastitis abscesses that were not drained with interventional radiological procedures. Puerperal mastitis healed with antibiotic therapy in a week. Seven granulomatous mastitis cases received steroid therapy after biopsy (Table 4). The granulomatous mastitis case is shown in Figure 1. Three locally advanced cancer cases were non-operable (Figure 2). There is a multidisciplinary breast council in our clinic. We evaluate breast patients before and after the operation in this council. In our clinic, a minimum of 150 and a maximum of 250 breast cancer operations are performed annually. In benign diseases, an average of 150 to 200 patients are operated on. With the COVID-19 pandemic, the number of patients who were diagnosed and operated on decreased.

**Table 1:** Seven patients referred for hormone therapy. ER, estrogen receptor; PR, progesterone receptor; +, axillary metastasis present; −, no axillary metastasis; HER2, human epidermal growth factor receptor 2

Age (years)	Tumor size (cm)	Receptors	Ki67 index (%)	Medication used	Follow-up time (days)	Tumor size change (cm)	Additional diseases	Axillary biopsy (fine-needle aspiration)
61	4	ER+, PR+, HER2−	20	Aromatase inhibitors (2.5 mg/day)	80	2.2	Diabetes mellitus and hypertension	+
63	1.8	ER+, PR+, HER2+	35	Aromatase inhibitors (2.5 mg/day)	68	1.5	Chronic heart failure	−
79	2.3	ER+, PR+, HER2−	15	Aromatase inhibitors (2.5 mg/day)	76	1.8	Ankylosing spondylitis	−
71	3.8	ER+, PR+, HER2+	25	Aromatase inhibitors (2.5 mg/day)	45	3.5	Coronary bypass surgery	−
70	4	ER+, PR+, HER2−	10	Aromatase inhibitors (2.5 mg/day)	38	3.4	None	+
68	3.2	ER+, PR-, HER2−	14	Aromatase inhibitors (2.5 mg/day)	42	2.9	Chronic renal failure and hypothyroidism	+
73	3.7	ER+, PR+, HER2−	30	Aromatase inhibitors (2.5 mg/day)	38	3.2	Diabetes mellitus, hypertension, and chronic heart failure	Suspicious

**Table 2:** Neoadjuvant treatment for patients. PET-CT SUV, positron emission tomography-computed tomography standardized uptake value; max., maximum; ER, estrogen receptor; PR, progesterone receptor; +, axillary metastasis present; HER2, human epidermal growth factor receptor 2

Age (years)	Tumor size (cm)	Receptors	Axillary biopsy (fine-needle aspiration)	PET-CT SUV max. value	Ki67 index (%)
45	2.5	ER−, PR−, HER2−	+	Tumor 17.6, axillary 14.6	45
60	2 and 4.5	ER−, PR−, HER2+	+	Tumor 11.2, axillary 10.8	30
54	5	ER+, PR+, HER2+	+	Tumor 8.6, axillary 7.3	15
57	3.5	ER−, PR+, HER2−	+	Tumor 9.8, axillary 11	45
58	2.2 and 1.7	ER+, PR+, HER2+	Suspicious pathology	Tumor 12.6, axillary 11.4	30
38	3.2 and 1.8	ER−, PR+, HER2+	+	Tumor 31.4, axillary 31.8	35
41	5	ER−, PR−, HER2−	+	Tumor 6.8, axillary 5.3	20
43	3.5	ER−, PR−, HER2−	+	Tumor 14, axillary 10	80

**Table 3:** Operated patients with malign lesions. USG, ultrasonography; MG, mammography; ER, estrogen receptor; PR, progesterone receptor; SNLB, sentinel lymph node biopsy; Preop., preoperatory; +, radiological technique performed; HER2, human epidermal growth factor receptor 2

Age (years)	Location	USG + MG	MRI	Neoadjuvant	Receptors	Ki67 (%)	Surgery	Stage	Additional illness
45	Top right outer	+	+	None	ER−, PR+, HER2−	20	Segmental mastectomy + SNLB	Stage 1B	Diabetes mellitus and hypertension
45	Lower right outer	+	+	None	ER+, PR+, HER2−	5	Segmental mastectomy + SNLB	Stage 2A	None
52	Upper left outer	+	+	Preop.	ER+, PR+, HER2+	30	Modified radical mastectomy	Stage 3A	None
32	Upper left middle	+	+	Preop.	ER−, PR−, HER2−	45	Segmental mastectomy + SNLB	Stage 2C	None
51	Top right outer	+	None	None	ER−, PR−, HER2−	20	Segmental mastectomy + SNLB	Stage 2A	None
38	Bottom left inner	+	+	None	ER+, PR+, HER2+	30	Segmental mastectomy + SNLB	Stage 1C	None
63	Bottom right inner	+	None	None	ER−, PR−, HER2+	15	Modified radical mastectomy	Stage 2A	Cirrhosis and congenital heart failure
43	Top left outer	+	+	Preop.	ER−, PR−, HER2−	60	Segmental mastectomy + axillary curettage	Stage 2C	Mix connective tissue disease
37	Right retroareolar	+	None	Preop.	ER−, PR−, HER2−	40	Modified radical mastectomy	Stage 2B	None

**Table 4:** Benign breast lesions.

Diagnosis	Number	Age range (years)	Interventional radiology	Treatment
Puerperal mastitis	5	18-36	Diagnosis with ultrasound	Antibiotic therapy: first-generation cephalosporin 2 g daily
Puerperal abscess	8	24-29	Abscess drainage with ultrasound guidance	Abscess drainage + antibiotics
Granulomatous mastitis	7	28-37	Drainage and biopsy with ultrasound guidance	Open drainage 3 cases + Prednol 16 mg-2 * 1/day after biopsy

## Discussion

Findings accumulated since the pandemic suggest that cancer patients are at a higher risk for COVID-19 infections and have higher morbidity and mortality than the general population [11]. In a study involving 28 cancer patients, it was observed that these patients had twice the risk of COVID-19 infections when compared to the general population [12]. The risks are elevated due to severe issues, such as patients who are inoperable or operable but at risk of widespread metastasis if left untreated. These patient groups have undergone surgical interventions or received alternative treatments to manage the disease [13].

During COVID-19, our hospital was declared a pandemic hospital, and many measures were taken. Outpatient clinic applications for elective patients were closed. All units of the hospital were filled with COVID-19 patients. Because the intensive care units were full, even the operating rooms where elective surgeries were performed were used as intensive care units. This caused all elective surgeries to almost come to a halt. Patients diagnosed with cancer and whose treatment had started preferred not to leave their homes out of fear of the pandemic. In this group of patients with suppressed immune systems, applications for neoadjuvant treatment decreased due to fear, and some of these patients died due to COVID-19.

On the other hand, patients who were examined for suspicious mass in their breasts could not receive outpatient clinic services, and the diagnosis process was delayed. Additionally, screening mammograms could not be performed. This primarily resulted in delays in the diagnosis of ductal carcinoma in situ (DCIS). Nevertheless, we know a correlation between DCIS cases with human epidermal growth factor receptor 2 (HER2) expression and ipsilateral breast cancer recurrence [14]. The delay in diagnosis and treatment decreased our encounter with invasive breast cancers in the post-pandemic period.

During the pandemic, breast cancer and benign breast diseases have become the group of diseases in which alternative surgery methods are preferred and surgical treatments are avoided as much as possible [15]. In this process, we had to surgically treat patients diagnosed with breast cancer who completed neoadjuvant therapy. Oncological treatments followed surgical treatments using the maximum delay times [16-18]. Hormonotherapy is especially recommended for patients with breast cancer who have not received chemotherapy in recent years or cannot be operated on due to their different diseases. During the pandemic, hormonotherapy was preferred over operation worldwide for hormone-positive patients [19-21]. We preferred aromatase inhibitors for our newly diagnosed postmenopausal patients who were suitable for hormonotherapy. We followed up with monthly physical examinations and ultrasound imaging. Finally, we prepared the patients who had achieved over a 40% response for surgery. 

In nod-positive patients with stage 1-2 tumors, the standard treatment was neoadjuvant chemotherapy [8,22,23]. Perhaps one of the approaches that did not change during the pandemic was to start these patients with chemotherapy. In this process, eight newly diagnosed patients were directed to medical oncology for neoadjuvant chemotherapy.

Although the ideal starting time for radiotherapy after breast conservation surgery is four to six weeks, publications state that it can be safely delayed for up to 20 weeks [24,25]. In this process, we have chosen to continue treating our patients who have already commenced radiotherapy. However, for those who have not yet started radiotherapy, we prefer to postpone their treatments.

Chemotherapy protocols are the first choice in local advanced metastatic cases [26]. Our approach to these cases was the same during the pandemic. Three cases were directed to oncology with the diagnosis of locally advanced breast cancer.

The most challenging patient group was stage 1-2 node-negative patients, in which surgery was the first choice despite all the risks. Although the number was not very high, we performed breast conservation surgery and sentinel lymph node biopsy in three patients. One patient did not want to receive radiotherapy, and her tumor was located in the retroareolar position, so we conducted a mastectomy. We operated under the condition that the COVID-19 polymerase chain reaction (PCR) test should be negative before the operation, and the surgery was performed in the negative pressure operating theaters.

The number of outpatients and follow-up patients was reduced due to the limited number of outpatient clinics and the fear of being sick. With the pandemic, benign breast diseases like fibroadenoma, papilloma, and cystic lesions were not followed up. While there are many publications on breast cancer, there was no publication or suggestion on benign breast diseases during the pandemic. Patients who presented at our hospital in emergency conditions received treatment for malignancy, mastitis, and abscesses. Patients who presented with abscess formation were monitored and treated with the assistance of interventional radiology. Patients with mastitis received antibiotic therapy. Granulomatous mastitis confirmed with biopsy was treated with prednisone, as the literature suggests. The surgical abscess was drained when necessary [27]. In summary, only those requiring urgent intervention were evaluated and treated for benign breast diseases [8,15].

## Conclusions

In conclusion, the pandemic situation in our country continues as it does worldwide. Although the transmission rates have decreased, we are still dealing with a fatal and contagious disease. Due to the COVID-19 pandemic, the treatment protocols for breast cancer and oncologic treatments have come to the fore, with a strong emphasis on minimizing surgical approaches. We acted by these protocols and recommendations. However, we believe that we need to plan new follow-up and treatment approaches to clarify how we can approach benign breast diseases that are not urgent.
